# Schiff base functionalized silica gel for simultaneous separation and preconcentration of Cu(II), Ni(II), and Cd(II) in pharmaceuticals and water samples

**DOI:** 10.3906/kim-2109-11

**Published:** 2021-12-06

**Authors:** Feyzullah TOKAY, Sema BAĞDAT

**Affiliations:** Department of Chemistry, Faculty of Science and Arts, Balıkesir University, Balıkesir, Turkey

**Keywords:** Solid phase extraction, Schiff base, water, pharmaceuticals, preconcentration, silica gel

## Abstract

A method for separation and preconcentration of Cu(II), Ni(II), and Cd(II) using N-N′-bis(5-methoxsalicylidene)-2-hydroxy-1,3-propanediamine modified silica gel was improved for aqueous samples and pharmaceuticals. Determination of the analytes was achieved by inductively coupled plasma optic emission spectrometry. The experimental conditions including pH value, sample volume, eluent type and volume, sorbent mass, sample, and eluent flow rates were optimized with univariate and multivariate optimization tools. The relative standard deviations of the method were 2.9% for Cu(II), 3.0% for Ni(II), and 3.3% for Cd(II) with recovery values between 98.8 ± 3.2–101.5 ± 3.0%. The limits of detection were found to be 62.4, 39.5, and 28.2 ng L^−1^ for Cu(II), Ni(II), and Cd(II), respectively. The accuracy of the suggested procedure was tested with the certified reference material (Certipur ICP multi-element standard solution IV) and addition-recovery experiments. The method was successfully applied to eye drop, anesthetic, serum, tap water, mineral water, and spring water samples.

## 1. Introduction

Accurate and precise quantification of pollutants has been a challenge and an important issue for analytical chemists. In addition to the importance and key roles of trace elements in biochemical reactions, it is known that they may be toxic even at trace concentration levels [1]. In recent years, the tremendous release of effluents from various industries led to environmental pollution. Therefore, the determination of trace metals is carried out systematically in various samples including food, environmental and pharmaceutics to monitor and/or designate the level of trace metal levels [2–5].

Flame atomic absorption spectrometry (FAAS) [6], electrothermal atomic absorption spectrometry (ETAAS) [7], atomic fluorescence spectrometry (AFS) [8], inductively coupled plasma optical emission spectrometry (ICP OES) [2] or mass spectrometry (ICP MS) [9] are the most used detection techniques for trace elements. Considering simultaneous detection of the analytes, ICP OES offers rapid, relatively cheaper, and accessible analysis. Unfortunately, limitations such as matrix interferences and low concentrations of analytes restrict the laboratory applications of these techniques. The most feasible way to overcome those limitations is to utilize separation and preconcentration methods for the removal of matrix effects and increase the concentration of target analyte(s). Consequently, considering the demand in the field, analytical chemists are interested in developing new methods [2, 10].

Separation and preconcentration methods including solid phase extraction (SPE) [2], liquid-liquid extraction (LLE) [11], cloud point extraction [12], precipitation [13], flotation [14], electrochemical deposition [15], membrane filtration [16] have been extensively used to separate and preconcentrate trace metals prior to detection. Among these methods, SPE is a simple and cost-effective method for separation and preconcentration of target analytes and overcomes many drawbacks such as usage of excessive solvent amount, laborious and time-consuming laboratory procedures. Additionally, high preconcentration factor, high recovery, ability to automate (to assemble with detection techniques for online analysis), high sample frequency, and eco-friendliness make SPE increasingly attractive and popular, which are also in good agreement with green chemistry applications.

Numerous bare or modified substances including silica gel, alumina, activated carbon, XAD resins, nano-materials, magnetic particles, polyurethane foam, and biosorbents that include high bonding affinity oxygen, nitrogen, and sulphur donor atoms have been utilized for separation and preconcentration of trace elements. According to reports, the resins that are modified using a chelating agent have triple benefits and functions: (i) sorption, (ii) ion exchange, and (iii) chelating [17–19]. A number of various previous reports showed that silica gel is preferred solid support due to its mechanical strength, thermal stability, larger surface area, and non-swelling features. Although the advantage of usability of silica gel is directly because of silanol groups, modification of the surface via organo-functional groups enhance the selectivity, binding capacity, and preconcentration factor [20].

Separation and preconcentration of the analyte(s) may be affected by various experimental conditions. The literature survey proved that the effect of variables was mostly exhibited using one factor at a time (OFAT) optimization [21]. Although this optimization seems simple, the number of experiments that need to be carried out is quite high. Moreover, the obtained data do not explain interactions between variables. However, utilization of experimental design such as central composite design (CCD) may simultaneously save time and explain the interactions between several variables with less number of experiments [6, 22].

The main objective of this study was to modify silica gel with N-N′-bis(5-methoxsalicylidene)-2-hydroxy-1,3-propanediamine (5MSHP) Schiff base and to use it as solid-phase extractant (Si-5MSHP) for simultaneous separation and preconcentration of Cu(II), Ni(II), and Cd(II) from pharmaceuticals water samples. Herein, we report a developed method for the first time for simultaneous separation and preconcentration of the target analytes from aqueous pharmaceuticals without any pretreatment. Determination of the analytes was achieved by ICP OES. The parameters effective on sorption and elution of the target cations such as pH and eluent type were optimized by OFAT, and sample volume, sorbent amount, flow rate, eluent volume, and concentration were optimized by CCD. The described methodology was successfully applied on various aqueous pharmaceuticals and water samples for the determination of Cu(II), Ni(II), and Cd(II).

## 2. Material and methods

### 2.1. Reagents and chemicals

All the reagents used were of analytical grade and used as received unless otherwise noted. The water used in all studies was obtained from the reverse osmosis system. The laboratory equipment used was kept overnight in 10% HNO_3_, rinsed with water, and dried at room temperature. The reagents 2-hydroxy-5-methoxy benzaldehyde and 2-hydroxy-1,3-propandiamine were purchased from Merck (Darmstadt, Germany) and Fluka (Munich, Germany), respectively. Silica gel (70–230 mesh) was received from Merck (Darmstadt, Germany). The desired pH value was adjusted using diluted HNO_3_ and NaOH (Merck, Darmstadt, Germany) solutions. Aqueous working solutions of Cu(II), Ni(II), and Cd(II) were prepared from nitrate salts (Merck, Darmstadt, Germany) at 1000 mg L^−1^ and diluted daily prior to use. The calibration curves for ICP OES measurements were established using the standard solutions in 3% HNO_3_ by dilution of individual Merck standard solutions of Cu(II), Ni(II), and Cd(II) (1000 mg element per liter, Darmstadt, Germany). On the other hand, a Merck multi element standard solution (1000 mg of each element per liter, Darmstadt, Germany) was utilized as standard reference material. The pharmaceutical aqueous samples eye drop, anesthetic, and serum were purchased from a pharmacy in Balıkesir, Turkey. Additionally, mineral and spring water samples were purchased from a local market and collected from Paşaköy, Balıkesir, Turkey, respectively.

### 2.2. Apparatus

Determination of the analytes was carried out using Perkin Elmer 7300 DV model ICP OES (Waltham, MA, USA). The operating parameters for the spectrometer were set as recommended by the manufacturer, and the operating conditions were given in Table 1. The emission lines were 327.393 nm, 231.604 nm, and 228.802 nm for Cu(II), Ni(II), and Cd(II), respectively. Characterization of Si-BSHP was implemented using PANalytical X Pert-Pro X-ray diffractometer (XRD) (Cu Kαλ = 1.54060 ˚A, 30 mA, 40 kV) (Malvern, UK) and Perkin Elmer Spectrum 65 Fourier Transform infrared attenuated total reflectance (FTIR-ATR) (Waltham, MA, USA) spectrometer. Separation and preconcentration studies were carried out with a funnel-tipped column that merged to Velp Scientifica SP311 peristaltic pump (Usmate, Italy) for flow control. Biosan ES-20 orbital shaker (Riga, Latvia), Sartorius TE214S electronic balance (Bradford, MA, USA), Thermo Scientific Orion 5 Star model pH meter (Waltham, MA, USA), and Elektro-mag M815 P centrifuge (İstanbul, Turkey) were used throughout the experiments.

### 2.3. Synthesize of 5MSHP and Preparation of Si-5MSHP

The Schiff base N-N′-bis(5-methoxsalicylidene)-2-hydroxy-1,3-propanediamine (5MSHP) was obtained by the condensation reaction of 2-hydroxy-5-methoxy benzaldehyde and 2-hydroxy-1,3-propandiamine as reported previously [23]. Accordingly, 0.9012 g (10 mmol) of 2-hydroxy-1,3-propandiamine in 40 mL ethanol solution (at 40 °C) was added on 3.0430 g (20 mmol) 2-hydroxy-5-methoxy benzaldehyde containing 40 mL ethanol solution (at 40 °C). Subsequently, the obtained yellowish compound was filtered, recrystallized, and used as a chelating agent.

In order to prepare Si-5MSHP sorbent, our previously reported process was followed. Primarily, a 10 g portion of purchased silica gel was washed with 50 mL of 0.5 mol L^−1^ HNO_3_ to remove any possible impurities. Afterwards, activated silica gel was filtered off and washed with purified water until neutralized. Finally, the activated silica gel was suspended in 50 mL acetone containing 50 mg 5MSHP for 2 h. The procedure was carried out under normal conditions. After the modification is completed, the sorbent was washed with purified water several times to remove any unsorbed 5MSHP residuals and dried at room temperature in a dust-free environment.

### 2.4. Configurable sample flow module

Column experiments were carried out using a funnel-tipped glass column (100 mm length, 10 mm inside diameter) with a glass frit resin support over the stopcock. A 0.75 g of Si-5MSHP portion was manually packed in the column throughout the simultaneous separation and preconcentration of target analytes. The bed height in the column was approximately 1 cm. A dropping funnel was installed over the column to prevent undulation during sample flow. After effective packaging, the column was fixed to the suction port of the peristaltic pump for configurable sample flow. The generated module was used for separation and preconcentration of Cu(II), Ni(II), and Cd(II) in column experiments.

### 2.5. Optimization strategy

The parameters pH and eluent type were optimized according to the one factor at a time procedure in batch experiments. On the other hand, a chemometric tool [24] was utilized to optimize variables for sorption (flow rate, volume, and amount of sorbent) and elution (flow rate, eluent volume, and concentration). As demonstrated in Table 2, the central composite design was performed in five levels to estimate the significance of the effects of variables for sorption and elution, separately. The total number of runs (N):


$$N=2^{f}+2f+N_{0}$$

where, *f* is number of variable and *N_0_* is the number of central points were 20 as given in Table 2. The mathematical relationship between three independent variables can be approximated by the second order polynomial model:


$$y=\beta_{0}+\sum_{i=1}^{n}\beta_{i}\;x_{i}+\sum_{i=1}^{n-1}\sum_{j=i+1}^{n}\beta_{ij}x_{i}x_{j}+\sum_{i=1}^{n}\beta_{ii}x_{i}^{2}$$

where y is response (dependent to recovery percentage), n is number of variables, *x_i_* and *x_j_* are notations for variables, *β_0_* is regression coefficient, *β_i_* is linear coefficient, *β_ij_* interanction coefficient, and *β_ii_* quadratic coefficients.

### 2.6. Solid phase extraction procedure

In order to survey the ability of the proposed methodology, the suggested procedure was applied for the determination of Cu(II), Ni(II), and Cd(II) in aqueous pharmaceuticals and water samples. A 35 mL portion of the aqueous sample was adjusted to pH = 4 with dilute HNO_3_ or NaOH solutions. Afterwards, the sample was passed through the sample flow module at 3.0 mL min^−1^ flow rate. The retained target analytes were eluted with 4.0 mL of 0.5 mol L^−1^ HNO_3_ at 2.6 mL min^−1^ flow rate. The eluate was collected in a test tube, and ICP OES was subsequently employed to determine the concentrations of target analytes. Purified water adjusted to pH = 4 with dilute HNO_3_ or NaOH solutions was subjected to described procedure as the reagent blank solution.

## 3. Results and discussion

### 3.1. Characterization of *N-N′-bis(5-methoxsalicylidene)-2-hydroxy-1,3-propanediamine* loaded silica gel

**Figure 1:**
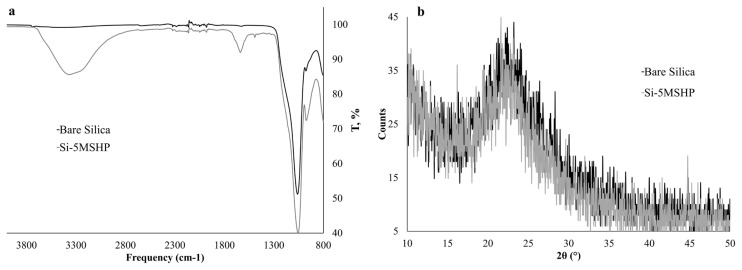
FT-IR spectrum (a) and XRD pattern (b) for bare and modified silica gel.

FT-IR is considered an efficient apparatus for tracking the changes in functional groups. Additionally, XRD is also frequently utilized for the identification of inorganic structures. The immobilization of silica gel was monitored using FT-IR and XRD, and the obtained spectra were given in Figure 1a and Figure 1b, respectively. In comparison to FT-IR spectrum of bare and Schiff base loaded silica gel, new peaks were observed in Figure 1a. The IR spectrum of the modified silica gel has some different peaks that correspond to ligand at 1490 cm^−1^ C-H bending and 1645 cm^−1^ C=N stretching.

According to the XRD pattern depicted in Figure 1b, a broad diffuse peak maxima is located at 21°, which is a well-known amorphous peak for silica [25]. Additionally, it was reported that organic moieties loaded on the inorganic structure may lead to a decrease in intensity due to surface coating. Correspondingly, there was a decrease in the intensity of the 5MSHP loaded silica gel.

Moreover, the amount of 5MSHP loaded on silica gel was determined according to the thermal desorption method. Mass of 5MSHP impregnated on silica gel was determined as 53.3 ± 3.4 mg g^−1^. Additionally, the covered 5MSHP mass was also given as mole as 148.0 ± 9.7 μmol g^−1^. The experimental results obtained from FT-IR, XRD, and thermal desorption experiments obviously showed that silica gel was successfully modified with 5MSHP.

### 3.2. Batch experiments

#### 3.2.1. Effect of pH value

**Figure 2:**
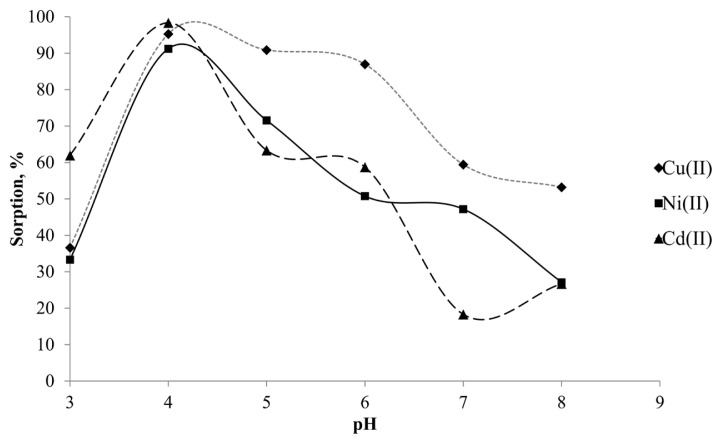
Effect of pH value on the sorption of target analytes.

An appropriate pH value may improve the sorption efficiency. Therefore, the effect of pH value on the retention of the target analytes on Si-5MSHP was examined at different pH values from 3 to 8. Considering the potential precipitation of metal ions in alkaline environment, pH values over 8 were not studied. Similarly, pH below 3 was also not examined due to the elution tendency of the analytes and possible degradation of the Schiff base in acidic media. For this purpose, 5 mL of standard solutions containing 150 ng of each analyte was treated with 0.5 g Si-5MSHP sorbent between pH = 3 and pH = 8. After 1 h agitation, the supernatant was pipetted and subjected to ICP OES analysis. As shown in Figure 2, the maximum extraction efficiency was obtained at pH = 4. The extraction efficiency was lower as expected at pH = 3, due to the competition between hydronium ions and target analytes toward Si-MSHP surface. On the other hand, at higher pH values, the possible anionic hydroxyl complexes of analytes, the sorption recoveries were not quantitative. Consequently, pH = 4 was selected and used for subsequent studies.

#### 3.2.2. Eluent choice

**Figure 3:**
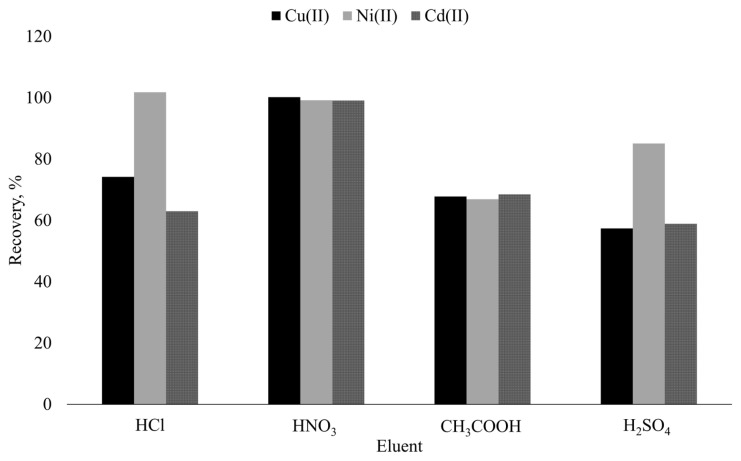
Effect of eluent type on desorption of target analytes.

The choice of suitable eluent is one of the significant factors for the separation and preconcentration of analytes using SPE. It is known that acids are effective in decomposition of metal complexes and strip the analytes from the solid support surface. Consideringly, the removal of the target from the surface of Si-5MSHP was tested using 5 mL of 0.5 mol L^−1^ HCl, HNO_3_, CH_3_COOH, and H_2_SO_4_ solutions. Due to the need of special instrumental accessories for ICP OES, organic solvents were out of assessment in selection of appropriate eluent types. The results given in Figure 3 emphasized that quantitative and simultaneous desorption of the analytes were only achievable with HNO_3_ solution. On the other hand, it should be underlined that HCl solution may be utilized for desorption of Ni, singly. Finally, HNO_3_ solution was selected as desorption reagent, and volume and concentration parameters were optimized in further experiments via CCD.

#### 3.2.3. Batch experiments vs. column studies

**Figure 4:**
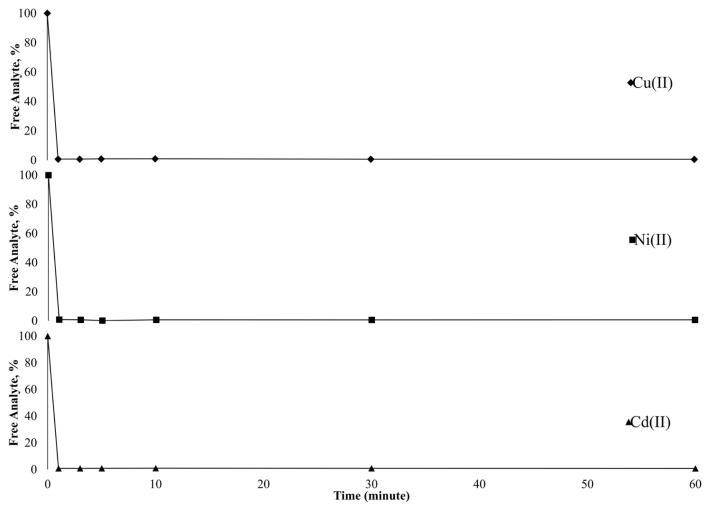
Time-dependent sorption behavior of target analytes.

Besides the advantages of solid-phase extraction procedure, column applications present some superiorities compared to batch experiments. In column applications, the sample solution is passed through solid support containing column via positive or negative pressure and the analytes retain on active sites of the sorbent. Then, the analytes are desorbed and may be subjected to detection technique in their current form. This application adds value to solid-phase extraction. Furthermore, speed, easy applicability, automation ability, and no need for experienced staff make column applications attractive. Consideringly, time-dependent sorption behavior of Cu(II), Ni(II), and Cd(II) was tested using 30 μg L^−1^ solutions with batch studies. The obtained results were depicted in Figure 4 as the graph of free analyte percentages versus time. The results clearly showed that free analyte content was below 1% in only 1 min for each analyte. The fast kinetic data make it possible to use column studies with the sorbent Si-5MSHP. Furthermore, this study also proved the stability of the retention of the analytes on the sorbent surface up to 60 min.

### 3.3. Column studies

#### 3.3.1. Optimization of sorption and elution variables

A central composite design was utilized in order to optimize the certain parameters for sorption and elution. For this purpose, 20 runs were set for the design. All the variables were investigated at 5 levels coded as −1.682, −1, 0, +1, and +1.682. The factors and notations were flow rate (F_S_), volume (V_S_), and amount of sorbent (m) for sorption and flow rate (F_E_), eluent volume (V_E_), and concentration (C). The order of the runs was tabulated in Table 2 and applied randomly to eliminate systematic errors. Additionally, the experimentally recorded response values were also given in Table 2 for the sorption and elution of target analytes, separately. The concentration of the target analytes was 30.0 μg L^−1^ in all CCD experiments.

The response values were fitted with second-order polynomial expression models including linear (F_S_, V_S_, m, F_E_, V_E_, C), polynomial (F_S_^2^, V_S_^2^, m^2^, F_E_^2^, V_E_^2^, C^2^), and cross (F_S_V_S_, F_S_m, V_S_m, F_E_V_E_, F_E_C, V_E_C) terms of variables. The equations can be expressed as


$$\begin{array}{c} y=0.3360+0.6403F_{s}-0.0346V_{s}+0.0810m+0.7069F_{s}^{2}-0.2792V_{s}^{2}-0.2577m^{2}+0.1074F_{s}V_{s}\\ -0.0636F_{s}m-0.0605V_{s}m \end{array}$$

for sorption of Cu(II),


$$\begin{array}{c} y=0.3649-0.0408F_{s}-0.0311V_{s}+0.0756m-0.0800F_{s}^{2}-0,0887V_{s}^{2}-0.0796m^{2}+0.0717F_{s}V_{s}\\ -0.0695F_{s}m-0.0189V_{s}m \end{array}$$

for sorption of Ni(II) and


$$\begin{array}{c} y=0.4988-0.0316F_{s}+0.0249V_{s}+0.0672m-0.1506F_{s}^{2}-0,1531V_{s}^{2}-0.1043m^{2}-0.0357F_{s}V_{s}\\ -0.0267F_{s}m+0.0809V_{s}m \end{array}$$

for sorption of Cd(II).

Similar to sorption variables, the factors for elution was fitted with second order polynomial equations as following:


$$\begin{array}{c} y=2.3373+0.0497F_{E}+0.0103V_{E}+0.0176C-0.6869F_{E}^{2}-0.7978V_{E}^{2}-0.7944C^{2}-0.0380F_{E}V_{E}\\ -0.0389F_{E}C+0.0556V_{E}C \end{array}$$

for elution of Cu(II),


$$\begin{array}{c} y=0.3589+0.9147F_{E}+0.5972V_{E}+0.6657C+0.2386F_{E}^{2}+0.2359V_{E}^{2}+0.2192C^{2}+1.1507F_{E}V_{E}\\ +1.0961F_{E}C+1.6071V_{E}C \end{array}$$

for elution of Ni(II) and

The partial derivatives of the abovementioned equations were equalized to zero in order to calculate the coded values of the variables. Subsequently, the coded values were converted to actual optimized figures of variables. The data analysis was carried out using Microsoft Excel. The coded and optimized figures were summarized in Table 3 for each target analyte.

Analysis of variance (ANOVA) was performed by Design Expert software for sorption (Table 4) and elution (Table 5) of the analytes. The confidence level of 95.0% was employed for linear, quadratic, and interaction effects of the variables. The factors and/or interactions were considered significant when *p* < 0.05. In this case, the results show that model variables are statistically significant in sorption of Cu(II) and elution of Ni(II) and Cd(II).

**Figure 5:**
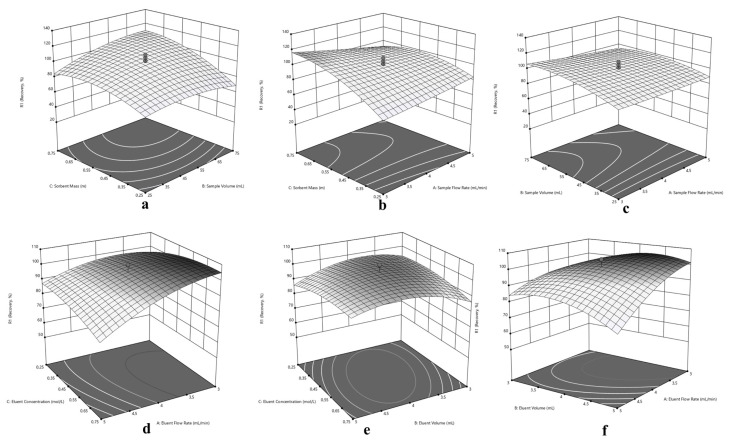
Response surfaces for separation and preconcentration of Cu(II): (a) sorbent mass (m) – sample volume (V_S_), (b) sorbent mass (m) – sample flow rate (F_S_), (c) sample volume (V_S_) - sample flow rate (F_S_), (d) eluent concentration (C_E_) – eluent flow rate (F_E_), (e) eluent concentration (C_E_) – eluent volume (V_E_), (f) eluent volume (V_E_) - eluent flow rate (F_E_).

**Figure 6:**
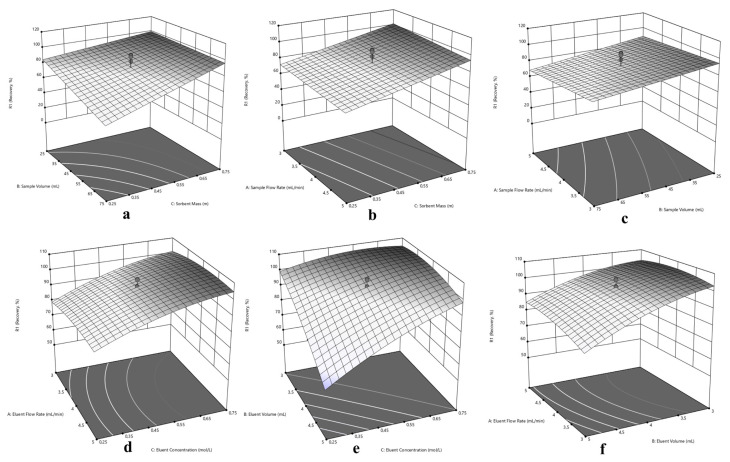
Response surfaces for separation and preconcentration of Ni(II): (a) sorbent mass (m) – sample volume (V_S_), (b) sorbent mass (m) – sample flow rate (F_S_), (c) sample volume (V_S_) - sample flow rate (F_S_), (d) eluent concentration (C_E_) – eluent flow rate (F_E_), (e) eluent concentration (C_E_) – eluent volume (V_E_), (f) eluent volume (V_E_) - eluent flow rate (F_E_).

**Figure 7:**
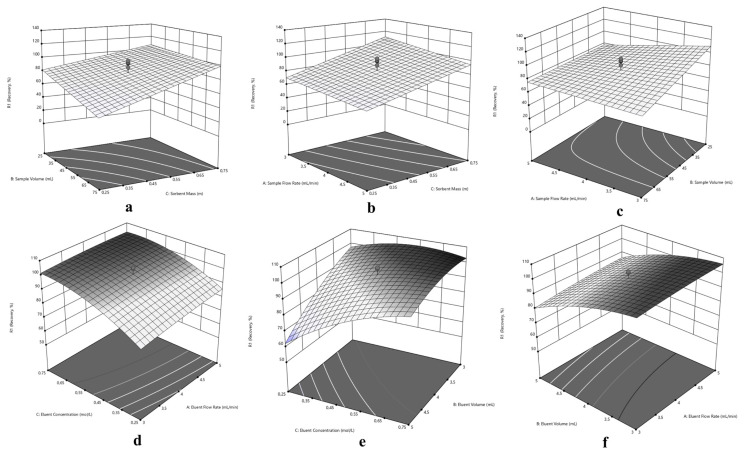
Response surfaces for separation and preconcentration of Cd(II): (a) sorbent mass (m) – sample volume (V_S_), (b) sorbent mass (m) – sample flow rate (F_S_), (c) sample volume (V_S_) - sample flow rate (F_S_), (d) eluent concentration (C_E_) – eluent flow rate (F_E_), (e) eluent concentration (C_E_) – eluent volume (V_E_), (f) eluent volume (V_E_) - eluent flow rate (F_E_).

Since the central composite design optimization has been applied for sorption and elution of Cu(II), Ni(II) and Cd(II) at the same time, a compromise has to be made in experimental conditions for simultaneous performance. In this purpose, three dimensional response surfaces were assessed to reveal the interactions between variables, visually. The 3D surface plots in Figure 5 for Cu(II), in Figure 6 for Ni(II) and in Figure 7 for Cd(II) indicate the interaction of two variables on the recovery at zero level of the other variables.

Typically, the interaction of V_S_ and m variables were depicted in Figure 5a, Figure 6a and Figure 7a for Cu(II), Ni(II) and Cd(II), respectively. Herein, it is observed that decrement in m regardless of V_S_ reduces recovery percentages for Cu(II). In terms of Ni(II), decrements in m and increment in V_S_ reduce recovery percentages. On the other hand, the highest recovery values were obtained for Cd(II) between 25 – 75 mL sample volume with 0.75 g sorbent mass. Regarding F_S_ and m, usage of sufficient sorbent mass (above 0.55 g), F_S_ has not significant effect on the quantitative recovery of Ni(II) (Figure 6b). On the other hand, according to Figure 5b and Figure 7b, the decrease in m and increase in F_S_ cause a decrease in the sorption percentage of Cu(II) and Cd(II). Considering the interaction between F_S_ and V_S_, at high V_S_ with low F_S_ satisfactory recoveries can be achieved for Cu(II) (Figure 5c). In terms of Ni(II) and Cd(II) (Figure 6c and Figure 7c), V_S_ about the value of 35 mL and F_S_ less than 4.5 mL min^−1^ present convincing results.

The interaction of elution parameters F_E_ and C_E_ were visualized in Figure 5d, Figure 6d and Figure 7d for Cu(II), Ni(II), and Cd(II), respectively. With regard to Cu(II) (Figure 5d), the concentration of HNO_3_ above 0.35 mol L^−1^ and F_E_ below 4.5 mL min^−1^ can provide quantitative recovery percentages. Contrary to Cu(II), when the concentration of HNO_3_ above 0.45 mol L^−1^, F_E_ has no significant effect on elution of Ni(II) and Cd(II). In the case of V_E_ and C_E_, the volume of HNO_3_ between 3.5–4.5 mL gave quantitative results for Cu(II) independent of C_E_ (Figure 5e). Concerning Ni(II) and Cd(II) (Figure 6e and Figure 7e, respectively), the concentration of HNO_3_ ≥0.45 mol L^−1^ present satisfactory elution percentages regardless of HNO_3_ volume. Regarding F_E_ and V_E_ parameters in elution of Cu(II) and Cd(II) (Figure 5f and Figure 7f, respectively), F_E_ ≤4 mL min^−1^ with volume of HNO_3_ between 3.5 and 5 mL and 3 and 5 mL, respectively, may provide quantitative elution. On the other hand, elution of Ni(II) (Figure 6f) may be achieved with V_E_ ≤ 4 mL, and the variable F_E_ has no significant effect.

Considering the overall data and simultaneous separation and preconcentration of Cu(II), Ni(II), and Cd(II), the performing conditions were selected as 3 mL min^−1^ sample flow rate, 35 mL sample volume, and 0.75 g sorbent mass for sorption cycle. Similarly, 2.6 mL min^−1^ eluent flow rate, 4 mL eluent volume and 0.50 mol L^−1^ eluent concentration was chosen for desorption cycle.

#### 3.3.2. Effect of potentially concomitants

Concomitant species in the aqueous solutions can disturb sorption of the target analytes. Therefore, the extraction efficiency may be reduced due to competition between the analytes and interferants. In order to investigate tolerance limits of various concomitants sorption behaviors of Cu(II), Ni(II), and Cd(II) were tested in selected conditions at 30 μg L^−1^ concentration. Considering the targeted sample variety, the selectivity of the proposed method was evaluated from different perspectives such as ionic strength (KNO_3_), presence of oxidative reagent (H_2_O_2_), and presence of donor atom containing species (thiourea and ethylenediaminetetraacetic acid, EDTA). To carry out an interference study, the sorption performance of the analytes was tested in the presence of 0.5 mol L^−1^ of each concomitant. The tolerance limits were defined as the concentration of concomitants that influenced analyte sorption more than ± 10%. Table 6 shows that the investigated concomitants have no significant effect on the separation and preconcentration of target analytes with the exception of Cu(II) sorption in the presence of donor atoms. Sorption percantages of Cu(II) were found as 86.0 ± 0.7% and 89.8 ± 3.0% in the presence of thiourea and EDTA, respectively. However, it should be noted that the ratios of concomitant and analytes concentrations are too high to be found in a natural sample. Consequently, it can be judged that separation and preconcentration of Cu(II), Ni(II), and Cd(II) may be selectively achieved with the proposed methodology from various aqueous sample types.

### 3.4. Figures of merit

The limits of detection (LODs) and the limits of quantification (LOQs) were calculated according to following equations based on IUPAC recommendation [26]: 3s_b_/m and 10s_b_/m (where m is the slope of calibration curve and s_b_ is the standard deviation of blank), respectively. LODs were found as 62.4 ng L^−1^ for Cu(II), 39.5 ng L^−1^ for Ni(II), and 28.2 ng L^−1^ for Cd(II). On the other hand, LOQs were calculated as 207.9, 131.5, and 93.9 ng L^−1^ for Cu(II), Ni(II), and Cd(II), respectively. The regression equations, correlation coefficients, and working ranges of the target analytes were displayed in Table 7. The calibration curves that were plotted by the method of least square after preconcentration of the analytes were found linear with >0.999 correlation coefficients in the working range. Considering the optimized working conditions, preconcentration factors of Cu(II), Ni(II), and Cd(II) were calculated as 10.4, 10.1, and 14.9, respectively by the ratio of sample volume to eluent volume. The sample throughput was 4.8 per h for selected separation and preconcentration conditions of each element. The evaluation of repeatability of the proposed method was assessed in terms of relative standard deviation (RSD). Herein, 10 repeated analyses of standard solutions containing 30.0 μg L^−1^ of each analyte were carried out according to recommended procedure. The RSD values were 2.9%, 3.0%, and 3.3% for Cu(II), Ni(II) and Cd(II), respectively. On the other hand, the obtained recovery values were 101.5 ± 3.0% for Cu(II), 99.9 ± 3.0% for Ni(II), and 98.8 ± 3.2% for Cd(II). Subsequently, the experimental data and the known amounts of the analytes were compared with the student’s *t*-test. The experimental *t* values were calculated as 1.581 for Cu(II), 0.105 for Ni(II), and 1.186 for Cd(II). The experimental *t* values were found less than the critical value of 2.262 at a 95% confidence level for 10 repeated analyses. The results indicated that there was no significant difference between experimental and theoretical concentrations.

The accuracy and feasibility of the proposed procedure were revealed by the analysis of independent multi element standard solution as CRM (Certipur ICP multi-element standard solution IV), which is diluted at two levels (7.5 μg L^−^1 and 30.0 μg L^−1^). The results (Table 8) were based on the average of the three replicates and in good agreement with the certified values. Additionally, a comparison of the obtained results and certified values was performed with the student’s *t*-test. The experimental *t* values were less than the critical *t* value (4.30), indicating that there is no systematic error at a 95% confidence level. Moreover, although the diluted CRM samples have included 20 other trace elements including transition metals except for the analytes, it can be underlined that there is no interference from these metals at the same levels as the analytes.

In terms of reusability of the prepared sorbent, considering the degradation of Schiff base structure in acidic conditions and the usage of 0.5 mol L^−1^ HNO_3_ in elution step, Si-5MSHP was utilized for one sorption-elution cycle. On the other hand, the thermal and mechanical stability of silica gel enables the modification process with 5MSHP plenty of times.

### 3.5. Real sample analysis

After validating the suggested procedure for separation and preconcentration of Cu(II), Ni(II) and Cd(II), we analysed various aqueous samples including eye drop, anesthetic, serum, tap water, mineral water and spring water. Assessment of the applicability of the procedure on natural and real samples was evaluated by the addition of known amounts of the target analytes and recovery. The obtained recovery values (Table 9) were 90.3–108.4% for Cu(II), 97.0–107.9% for Ni(II) and 94.3–109.3% for Cd(II) which are relatively acceptable. It is obvious from the results that the suggested procedure is not affected by the investigated matrixes. The unspiked samples were also analysed in triplicate, and the results were tabulated in Table 9. The detected concentrations were between 14.39 ± 0.68–19.02 ± 1.31 μg L^−1^ for Cu(II) and 0.81 ± 0.04–1.18 ± 0.14 μg L^−1^ for Cd(II) in water samples. Additionally, the concentration of Ni(II) was only detected in mineral water as 0.33 ± 0.01 μg L^−1^. The results obtained from the analysis of water samples were below the allowed limits by Turkish legislation. On the other hand, the detected amounts of Cu(II), Ni(II), and Cd(II) in eye drop, anesthetic, and serum samples were below the allowed limits by the United States Pharmacopeia (USP) and European Pharmacopeia (EP).

### 3.6. Comparison of the suggested procedure

A comparison of the proposed separation and preconcentration methodology with the other those reported in literature is tabulated in Table 10. It can be clearly seen that the suggested procedure exhibit satisfactory repeatability with comparable or mostly better LODs for the target analytes. One of the highlights of the study is the ability of simultaneous preconcentration and detection, which is not applicable for those. Moreover, it may be an encouraging and guiding reference for the analysis of pharmaceutical samples with solid-phase extraction, which is limited in the literature.

## 4. Conclusion

In this work, a Schiff base functionalized silica gel Si-5MSHP was prepared and used for separation and preconcentration of Cu(II), Ni(II), and Cd(II) from aqueous samples including water samples and pharmaceuticals prior to ICP OES detection. To our knowledge, this is the first reported method that covers simultaneous separation and preconcentration of Cu(II), Ni(II), and Cd(II) from aqueous pharmaceuticals without pretreatment. We think that preparation of the sorbent under atmospheric conditions is an advantage, and it provides superiority compared to sorbents prepared using consumables such as argon and nitrogen.

The efficiency of Si-5MSHP as sorbent was investigated using batch and column experiments via univariate and multivariate optimizations. The analytical performances of the methodology reported in this work including LOD, repeatability, sampling frequency, and preconcentration factor were found comparable with the literature. There was no interfering effect of KNO_3_, H_2_O_2_, EDTA, and thiourea in terms of ionic strength, oxidative reagent, and chelating agent, respectively on separation and preconcentration of the target analytes. The method was validated by the analysis of a CRM of multielement standard solution. Additionally, the method was successfully applied on eye drop, anesthetic, serum, tap water, mineral water, and spring water samples. The proposed system is simple, cost-effective, rapid, and analyst friendly for separation and preconcentration of the target analytes.

## Figures and Tables

**Table 1:** Operating conditions for ICP OES.

Torch viewing	*Axial*	Recalibration system	*Hg lamp*
RF power	*1300 Watts*	Nebulizer	*Cross flow*
Spray Chamber	*Glass cyclonic spray chamber*	Plasma gas flow	*15 L min* *^−1^*
Auxiliary gas flow	*0.2 L min* *^−1^*	Nebulization gas flow	*0.8 L min* *^−1^*
Sample flow rate	*1.5 mL min* *^−1^*	Delay time	*60 s*
Wash rate	*1.5 mL min* *^−1^*	Wash time	*30 s*

**Table 2:** Variables and experimental design matrix for CCD

Variables				Notation	Levels				
for sorption (S)					−α (−1.682)	Low (−1)	Central (0)	High (+1)	+α (+1.682)
Flow Rate (mL min^−1^)				F_S_	2.318	3	4	5	5.682
Sample Volume (mL)				V_S_	8.0	25.0	50.0	75.0	92.0
Amount of Sorbent (g)				m	0.08	0.25	0.50	0.75	0.92
for elution (E)									
Flow Rate (mL min^−1^)				F_E_	2.318	3	4	5	5.682
Eluent Volume (mL)				V_E_	2.318	3	4	5	5.682
Eluent Concentration (mol L^−1^)				C	0.08	0.25	0.50	0.75	0.92
Run	Variables			Response (S)			Response (E)		
	F_S_, F_E_	V_S_, V_E_	m, C	Cu(II)	Ni(II)	Cd(II)	Cu(II)	Ni(II)	Cd(II)
1	−1	−1	−1	0.0399	0.0880	0.1472	0.1105	0.2353	0.4203
2	+1	−1	−1	0.0727	0.0860	0.0455	0.0981	1.9960	1.2977
3	−1	+1	−1	0.0546	0.0575	0.0512	0.0273	0.0222	0.0243
4	+1	+1	−1	0.0491	0.0201	0.0212	0.0252	0.0257	0.0291
5	−1	−1	+1	0.7250	0.6383	0.0254	0.0413	0.2369	0.2721
6	+1	−1	+1	0.0358	0.0360	0.0317	0.0357	0.0223	0.3638
7	−1	+1	+1	0.0301	0.2098	0.4675	0.3430	0.0923	0.1582
8	+1	+1	+1	0.2382	0.2166	0.1163	0.0231	10.8402	0.2196
9	0	0	0	0.1057	0.2400	0.2075	0.0811	0.1049	0.2419
10	−1.682	0	0	0.0968	0.0414	0.0274	0.0491	0.0830	0.1031
11	+1.682	0	0	5.5659	0.0872	0.0543	0.6548	0.1991	2.7273
12	0	−1.682	0	0.0330	0.0638	0.0534	0.0361	0.2323	0.3988
13	0	+1.682	0	0.0501	0.0157	0.0140	0.0403	0.0346	0.0346
14	0	0	−1.682	0.0149	0.0108	0.0106	0.0305	0.0328	0.0283
15	0	0	+1.682	0.1897	0.1200	0.3332	0.0651	0.1395	3.8943
16	0	0	0	0.6893	0.3299	0.3777	11.7521	0.7612	0.4292
17	0	0	0	0.2324	0.1053	0.3833	0.2166	0.3130	0.3379
18	0	0	0	0.2022	0.6440	0.4580	0.2075	0.3676	0.3165
19	0	0	0	0.4354	0.5009	0.6725	1.6663	0.6447	0.7562
20	0	0	0	0.1818	0.3947	0.9046	0.1143	0.2669	0.2394

**Table 3:** Optimal operation conditions for separation and preconcentration of the analytes.

Variables	Unit	Analyte		
Sorption		Cu(II)	Ni(II)	Cd(II)
F_S_ (Sample Flow Rate)	mL min^−1^	3.58	3.01	3.83
V_S_ (Sample Volume)	mL	41.56	32.98	55.34
m (Sorbent Mass)	g	0.56	0.75	0.61
Elution				
F_E_ (Eluent Flow Rate)	mL min^−1^	4.04	4.44	2.58
V_E_ (Eluent Volume)	mL	4.00	3.25	3.71
C (Eluent Concentration)	mol L^−1^	0.50	0.40	0.33

**Table 4:** Analysis of variance (ANOVA) for CCD optimization of sorption step.

	Cu(II)					Ni(II)					Cd(II)				
Source	Sum of Squares	df	Mean Square	F-value	p-value	Sum of Squares	df	Mean Square	F-value	p-value	Sum of Squares	df	Mean Square	F-value	p-value
**Model**	6157.71	9	684.19	4.87	0.0106	3624.69	3	1208.23	2.68	0.0822	8126.51	6	1354.42	2.04	0.1326
A	103.30	1	103.30	0.7345	0.4115	101.98	1	101.98	0.2258	0.6411	650.11	1	650.11	0.9786	0.3406
B	421.96	1	421.96	3.00	0.1139	807.18	1	807.18	1.79	0.1999	1447.25	1	1447.25	2.18	0.1638
C	2404.27	1	2404.27	17.10	0.0020	2715.54	1	2715.54	6.01	0.0261	4991.68	1	4991.68	7.51	0.0168
AB	73.56	1	73.56	0.5230	0.4861						699.71	1	699.71	1.05	0.3235
AC	671.14	1	671.14	4.77	0.0538						17.61	1	17.61	0.0265	0.8732
BC	422.65	1	422.65	3.01	0.1137						320.16	1	320.16	0.4819	0.4998
A^2^	3.92	1	3.92	0.0278	0.8708										
B^2^	611.64	1	611.64	4.35	0.0636										
C^2^	1561.90	1	1561.90	11.11	0.0076										
**Residual**	1406.34	10	140.63			7225.36	16	451.58			8636.02	13	664.31		
Lack of Fit	1251.08	5	250.22	8.06	0.0195	7182.22	11	652.93	75.68	< 0.0001	8584.98	8	1073.12	105.14	< 0.0001
Pure Error	155.26	5	31.05			43.14	5	8.63			51.03	5	10.21		
**Cor Total**	7564.05	19				10850.05	19				16762.53	19			

A: Sample Flow Rate; B:Sample Volume; C: Sorbent Mass

**Table 5:** Analysis of variance (ANOVA) for CCD optimization of elution step

	Cu(II)					Ni(II)					Cd(II)				
Source	Sum of Squares	df	Mean Square	F-value	p-value	Sum of Squares	df	Mean Square	F-value	p-value	Sum of Squares	df	Mean Square	F-value	p-value
**Model**	2716.79	9	301.87	2.74	0.0663	4252.49	9	472.50	33.06	< 0.0001	3843.68	9	427.08	43.41	< 0.0001
A	1094.51	1	1094.51	9.93	0.0103	62.74	1	62.74	4.39	0.0626	56.37	1	56.37	5.73	0.0377
B	1.59	1	1.59	0.0145	0.9067	1669.26	1	1669.26	116.79	< 0.0001	1522.44	1	1522.44	154.75	< 0.0001
C	0.1413	1	0.1413	0.0013	0.9721	1569.68	1	1569.68	109.83	< 0.0001	1287.31	1	1287.31	130.85	< 0.0001
AB	226.59	1	226.59	2.05	0.1822	34.66	1	34.66	2.43	0.1505	5.07	1	5.07	0.5154	0.4893
AC	162.24	1	162.24	1.47	0.2530	0.6585	1	0.6585	0.0461	0.8344	10.44	1	10.44	1.06	0.3273
BC	85.92	1	85.92	0.7791	0.3981	502.81	1	502.81	35.18	0.0001	402.14	1	402.14	40.88	< 0.0001
A^2^	413.40	1	413.40	3.75	0.0816	90.28	1	90.28	6.32	0.0307	8.32	1	8.32	0.8461	0.3793
B^2^	778.60	1	778.60	7.06	0.0240	212.24	1	212.24	14.85	0.0032	206.08	1	206.08	20.95	0.0010
C^2^	138.16	1	138.16	1.25	0.2892	187.27	1	187.27	13.10	0.0047	405.68	1	405.68	41.24	< 0.0001
**Residual**	1102.68	10	110.27			142.92	10	14.29			98.38	10	9.84		
Lack of Fit	979.87	5	195.97	7.98	0.0199	33.76	5	6.75	0.3092	0.8882	46.35	5	9.27	0.8908	0.5490
Pure Error	122.81	5	24.56			109.17	5	21.83			52.03	5	10.41		
**Cor Total**	3819.48	19				4395.42	19				3942.06	19			

A: Eluent Flow Rate; B:Eluent Volume; C: Eluent Concentration

**Table 6:** Effect of potential concomitants

Potential Sorption Blocker*	Sorption, %		
	Cu(II)†	Ni(II)†	Cd(II)†
H_2_O_2_	93.2 ± 1.9	98.2 ± 4.1	92.9 ± 0.3
KNO_3_	98.8 ± 2.7	91.5 ± 1.5	91.0 ± 0.2
Thiourea	86.0 ± 0.7	91.6 ± 0.5	98.5 ± 0.4
EDTA	89.8 ± 3.0	91.1 ± 1.8	92.1 ± 0.3

* 0.5 mol L^−1^;

† 30 μg L^−1^

**Table 7:** Regression equations and characteristics of calibration curves

Analyte	Calibration equations	R^2^	Working Range (μg L^−1^)
Cu(II)	*y* = 2222.82*x* + 12.348	0.99998	0.21–50.00
Ni(II)	*y* = 51697.82*x* + 12.27	0.99996	0.13–50.00
Cd(II)	*y* = 828.976*x* + 2.884	0.99996	0.09–50.00

**Table 8:** Analysis of certified reference material

	Analyte		
	Cu(II)	Ni(II)	Cd(II)
Certified Value^1^ (μg L^−1^)	7.5	7.5	7.5
Found Value^1^ (μg L^−1^)	7.9 ± 0.6	7.4 ± 0.6	7.6 ± 0.7
Recovery, %	105.2	98.6	101.5
RSD, %	7.5	7.7	8.9
*t*_experimental_	1.15	0.29	0.25
Certified Value^2^ (μg L^−1^)	30.0	30.0	30.0
Found Value^2^ (μg L^−1^)	30.0 ± 1.3	28.9 ± 1.0	29.5 ± 0.5
Recovery, %	100.0	96.3	98.4
RSD, %	4.5	3.4	1.6
*t*_experimental_	1.3×10^−4^	1.90	1.73

**Table 9:** Results for the analysis of aqueous samples and pharmaceuticals

Sample	Added (μg L^−1^)	Cu(II)		Ni(II)		Cd(II)	
		Found (μg L^−1^)	Rec, %	Found (μg L^−1^)	Rec, %	Found (μg L^−1^)	Rec, %
Eye Drop	0.00	14.55 ± 0.74	-	nd*	-	1.16 ± 0.02	-
	1.00	15.64 ± 0.46	108.4	1.04 ± 0.13	103.6	2.12 ± 0.09	96.4
Anesthetic	0.00	7.26 ± 0.28	-	nd	-	0.41 ± 0.06	-
	1.00	8.27 ± 0.58	101.6	0.97 ± 0.06	97.0	1.41 ± 0.15	101.3
Serum	0.00	9.71 ± 0.87	-	-	-	1.51 ± 0.08	-
	1.00	10.62 ± 1.49	90.3	-	-	2.49 ± 0.13	97.7
Mineral Water	0.00	14.39 ± 0.68	-	0.33 ± 0.01	-	1.18 ± 0.14	-
	1.00	15.35 ± 1.09	95.4	1.37 ± 0.11	103.9	2.28 ± 0.08	109.3
Tap Water	0.00	18.80 ± 2.10	-	nd*	-	1.01 ± 0.06	-
	1.00	19.88 ± 1.92	107.0	1.08 ± 0.05	107.9	1.95 ± 0.10	94.3
Spring Water	0.00	19.02 ± 1.31	-	nd*	-	0.81 ± 0.04	-
	1.00	20.03 ± 0.35	101.1	1.07 ± 0.09	107.4	1.76 ± 0.06	94.8

* LOD values:

**Table 10:** Comparison of the proposed procedure.

Sample	Chelating Agent	Solid Support	Application Type of SPE	Analyte(s)†	Eluent	Detection Technique	Simultaneous Preconcentration/detection	LOD (μg L^−1^); (ng L^−1^)^*^	RSD, %	Ref
Water, Fertilizers, Pharmaceuticals	2,6- dimethyl-morpholine dithiocarbamate	Amberlite XAD-4	Column	Cu(II), Ni(II)	8.0 mL of 0.5 mol L^−1^ HNO3 (in acetone)	UV-Vis Spectrophotometry	+ / +	Cu:11.2; Ni:1.37	3.6–4.8	[4]
Water	o-cresolphthalein complexone	Multiwalled carbon nanotubes	Column	Cu(II), Ni(II)	10 mL of 2 mol L^−1^ HNO_3_	FAAS	+ / −	Cu:1.64; Ni:5.68	-	[17]
Water and Fertilizers	1,3,4-thiadiazole-2,5-dithiol	Activated carbon	Pipette tip	Cu(II), Ni(II), Cd(II)	5 mL of 3 mol L^−1^ HNO_3_	FAAS	+ / −	Cu:2.0; Ni:2.7; Cd:1.3	3.0–4.4	[18]
Water, black tea, diet supplements	Pyrocatechol violet	Magnetic graphene oxide	Dispersive magnetic	Cu(II)	2 mL of 2 mol L^−1^ HNO_3_	FAAS	+ / −	Cu:4.0	4.93	[19]
Water	N,N′-bis(salicylidene) phenylene-1,3-diamine	Silica gel	Column	Cu(II), Ni(II), Cd(II)	5 mL of 1 mol L^−1^ HNO_3_	FAAS	+ / −	Cu^*^:5 Ni^*^:4.7; Cd^*^:3	1.50–3.43	[20]
Water, Food Samples	*Pleurotus ostreatus*	Magnetic iron oxide nanoparticles	Column	Ni(II)	5 mL of 1 mol L^−1^ HCl	ICP OES	+ / +	Ni:0.019	2.3	[27]
*Water, Pharmaceuticals*	*N-N′-bis(5-methoxsalicylidene)-2-hydroxy-1,3-propanediamine*	*Silica gel*	*Column*	*Cu(II), Ni(II), Cd(II)*	*4 mL of 0.5 mol L* *^−1^* * HNO* *_3_*	*ICP OES*	+ / +	*Cu* ^*^ *:* *Ni* ^*^ *:39.5; Cd* ^*^ *:28.2*	*2.9–3.3*	*This work*

† only the target analytes were evaluated
